# Trends in cervical cancer incidence in sub-Saharan Africa

**DOI:** 10.1038/s41416-020-0831-9

**Published:** 2020-04-27

**Authors:** Elima Jedy-Agba, Walburga Yvonne Joko, Biying Liu, Nathan Gyabi Buziba, Margaret Borok, Anne Korir, Leo Masamba, Shyam Shunker Manraj, Anne Finesse, Henry Wabinga, Nontuthuzelo Somdyala, Donald Maxwell Parkin

**Affiliations:** 1 International Research Center of Excellence, Institute of Human Virology, Abuja, Nigeria; 2 Clinical Trials Service Unit, Nuffield Department of Population Health, University of Oxford, Oxford, UK; 3 African Cancer Registry Network, Prama House, 267 Banbury Road, Oxford, UK; 4 Eldoret Cancer Registry, Moi University School of Medicine, Eldoret, Kenya; 5 Zimbabwe National Cancer Registry, University of Zimbabwe College of Health Sciences, Harare, Zimbabwe; 6 Nairobi Cancer Registry, Kenya Medical Research Institute, Nairobi, Kenya; 7 University of Malawi College of Medicine and Queen Elizabeth Central Hospital Cancer Unit, Blantyre, Malawi; 8 Mauritius National Cancer Registry, Mauritius Institute of Health, Pamplemousses, Mauritius; 9 Seychelles National Cancer Registry, Ministry of Health, Victoria, Seychelles; 10 Department of Pathology, College of Health Sciences, Makerere University, Kampala, Uganda; 11 Eastern Cape Cancer Registry, Burden of Disease Research Unit, South African Medical Research Council, Cape Town, South Africa; 12 International Agency for Research on Cancer, Lyon, France

**Keywords:** Cancer epidemiology, Epidemiology

## Abstract

**Background:**

Cervical cancer is the second most common cancer and the leading cause of cancer death in women in sub-Saharan Africa (SSA).

**Methods:**

Trends in the incidence of cervical cancer are examined for a period of 10–25 years in 10 population-based cancer registries across eight SSA countries (Gambia, Kenya, Malawi, Mauritius, Seychelles, South Africa, Uganda and Zimbabwe). A total of 21,990 cases of cervical cancer were included in the analyses.

**Results:**

Incidence rates had increased in all registries for some or all of the periods studied, except for Mauritius with a constant annual 2.5% decline. Eastern Cape and Blantyre (Malawi) registries showed significant increases over time, with the most rapid being in Blantyre (7.9% annually). In Kampala (Uganda), a significant increase was noted (2.2%) until 2006, followed by a non-significant decline. In Eldoret, a decrease (1998–2002) was followed by a significant increase (9.5%) from 2002 to 2016.

**Conclusion:**

Overall, cervical cancer incidence has been increasing in SSA. The current high-level advocacy to reduce the burden of cervical cancer in SSA needs to be translated into support for prevention (vaccination against human papillomavirus and population-wide screening), with careful monitoring of results through population-based registries.

## Background

In 2018 it was estimated that there were about 570,000 new cases of cervical cancer (CC) worldwide, with 80% of these cases occurring in low- and middle-income countries.^1^ CC is the fourth most frequently diagnosed cancer of women worldwide, and the most common cancer in half (23/46) of the countries of sub-Saharan Africa (SSA) (although second in frequency to breast cancer overall).^1^ The global burden of CC is unevenly distributed worldwide and women in SSA are disproportionately affected with higher incidence and mortality rates than in any other region of the world. Southern Africa reports the highest age standardised incidence rate (ASR) of CC worldwide (43.1 per 100,000).^2^ In a recent 11-country study, survival from cancer of the cervix in SSA is poor—33% at 5 years post diagnosis (Sengayi-Muchengeti, personal communication). In 2018, 21.7% of all cancer deaths in SSA women were attributed to CC, making it the most common cause of cancer death in the region.^2^

In developed countries, such as the United Kingdom (UK) and the United States (US), the incidence of CC has fallen dramatically since the 1960s, owing to the implementation of population-wide screening programmes, cytology based initially, using human papillomavirus (HPV) DNA testing more recently.^3,4^ In contrast, the incidence of CC in developing countries continues to rise due to the absence of effective population-level screening programmes, poor awareness about prevention, inequitable access to health services, poverty and low socioeconomic status.^5–7^

Ginsburg et al.^7^ reviewed the burden of breast and CC in 2012, with an emphasis on global trends in incidence, mortality and survival. Despite improving knowledge about cancer in the region, data on incidence and mortality trends from many SSA countries remain limited owing to the lack of cancer registries having data of consistent quality for long time periods, as well as inadequate vital statistics systems in the region,^8^ although a few studies conducted in individual SSA countries have been published.^9–13^

The African Cancer Registry Network (AFCRN), through collaborations with its member population-based cancer registries in Africa, has contributed to improving cancer registration on the continent, and through its activities has generated data that can be used to estimate the burden of various cancers in SSA, with implications for cancer control in the region.^8^ In this present study, we investigate trends of CC incidence in ten cancer registries in three SSA regions, for a period between 10 and 25 years, to provide important information for the development of CC prevention and control strategies, and as a benchmark for monitoring of the effectiveness of such programmes, in a setting that bears a significant proportion of the worldwide burden of CC.^14^

## Materials and methods

Ten population-based cancer registries in eight countries, members of AFCRN, were included in the study: The Gambia, Kenya (Eldoret and Nairobi), Malawi (Blantyre), Mauritius, Seychelles, South Africa (Eastern Cape), Uganda (Kampala) and Zimbabwe (Bulawayo and Harare). All 10 are population-based, recording data in defined populations whose composition by age, sex and ethnic group is known. The methods of data collection, validation and storage of these registries are described elsewhere.^8^

The registries selected for the study were those that could provide estimates of the incidence of cancer of the cervix of consistent quality for periods of 10 or more years. Quality of the registration process was evaluated as described in Chapter 3 of ref. ^8^ Cases of CC (ICD-10 C53) and uterus unspecified (C55) were abstracted from the registry databases, along with the estimated populations at risk by age group and sex (and ethnicity, where appropriate). Population data were derived from census estimates, and intercensal estimates were calculated assuming an exponential growth rate between censuses. Annual age-specific, crude and age standardised rates were calculated, with age standardisation carried out by the direct method using the ‘world standard population’.^15^ As well as trends in the numbers (and rates) of cases of cancer of the cervix, we examined the rates of C55 (uterus NOS) to determine if there had been any temporal changes, suggesting differential misallocation of CCs to this category in these registries over time.

For those datasets/periods retained, we investigated trends in annual age standardised rates, fitting regression lines to determine whether the trends (best fit of the regression) were best explained as linear, exponential or polynomial. For registries for which there was a poor fit with a single trend (*R*^2^ < 0.5), due to a change during the period examined, we analysed trends in incidence using the Joinpoint Regression Program version 4.7.0.0^16^ developed by the US National Cancer Institute (NCI). The point(s) (years) at which statistically significant change(s) in trends occurred are identified by the Joinpoint regression. The average rate of change (annual percent change) in each trend segment was calculated using a Monte Carlo permutation method.^17^

For two datasets with 25-year periods available (Harare and Kampala), we also examined incidence rates by 5-year time periods, and present age-specific rates by time period, and birth cohort.

We calculated some conventional indicators of data quality^18,19^—the percentage of cases with morphological verification (histology or cytology) of diagnosis (MV%) and the percentage of cases registered by death certificate only (DCO%)—for the time periods under review. Results are presented for the individual cancer registries.

## Results

A total of 21,990 cases of CC were registered and included in this study from 10 population-based cancer registries across eight SSA countries. They are shown in Table 1, grouped according to the regions of SSA as defined by the United Nations. Of the 10 registries, Mauritius, Seychelles and The Gambia had national coverage, Eastern Cape covered a rural area and the rest covered populations that are predominantly urban. Bulawayo registry, active in the 1960s,^20^ was reactivated recently after a gap of 40 years, with rates available for 2012–2015. For Harare (Zimbabwe) we report a 25-year period (1991–2015), with the omission of 3 years (2007–2009) for which it was known that registration was incomplete (due to problems with the medical services during the economic crisis in those years).^12^ For Kampala, registration was incomplete for 2014, and the rates for the 4-year period (2010–2013) were taken to represent those for 2010–2014. Table 1 shows for each registry the average annual percentage change (AAPC) in the ASRs over the whole time period. Incidence rates have increased, at least for part of the period studied, for all except Mauritius, where a statistically significant decline of 2.5% per year was seen. The highest average annual increase was reported in Blantyre (Malawi) (7.9%). All the registries, with the exception of Blantyre and Kampala, reported >70% MV% of cases (Table 1).

**Table 1:** Cervical cancer cases (C53) by time period, total number of cases and most valid basis of diagnosis by cancer registry and region of SSA.

SSA region	Cancer registry	Population coverage	Time period	No. of cases	DCO (%)^a^	MV (%)^b^	AAPC (95% CI)
East Africa	Kenya, Eldoret	Urban	1998–2016	1081	1	91	2.6 (−9.0; 15.8)
	Kenya, Nairobi	Urban	2003–2014	3012	3	80	−1.5 (−4.7; 1.8)
	Malawi, Blantyre	Urban	1994–2010	2338	0.4^c^	52	7.9 (5.6; 10.2)
	Mauritius	National	2001–2015	1348	0	96	−2.5 (−3.8, −1.2)
	Seychelles	National	2004–2015	103	0	98	N/A^d^
	Uganda, Kampala	Urban	1990–2013	3519	0	63	1.3 (−0.7; 3.4)
	Zimbabwe, Bulawayo	Urban	1963–1972; 2012–2015	1434	0^e^	79	N/A
	Zimbabwe, Harare	Urban	1991–2006; 2010–2015	4515	6	78	1.2 (−0.2; 2.5)
Western Africa	The Gambia	National	1986–2014	1752	0	72	2.6 (−1.2; 6.5)
Southern Africa	South Africa, Eastern Cape	Rural	1998–2016	2888	0	78	3.5 (1.8; 5.3)

^a^DCO: Cases registered based on information contained on a death certificate only.

^b^MV: Cases for which diagnosis was based on cytology, haematology or histopathology.

^c^There were nine cases of DCO only in the entire period under review constituting only 0.38%.

^d^Number of cases by year were too few hence rates were calculated by time period and not annually.

^e^There were four cases of DCO only in the entire period under review in Bulawayo, which constituted only 0.14%.

**Fig. 1:**
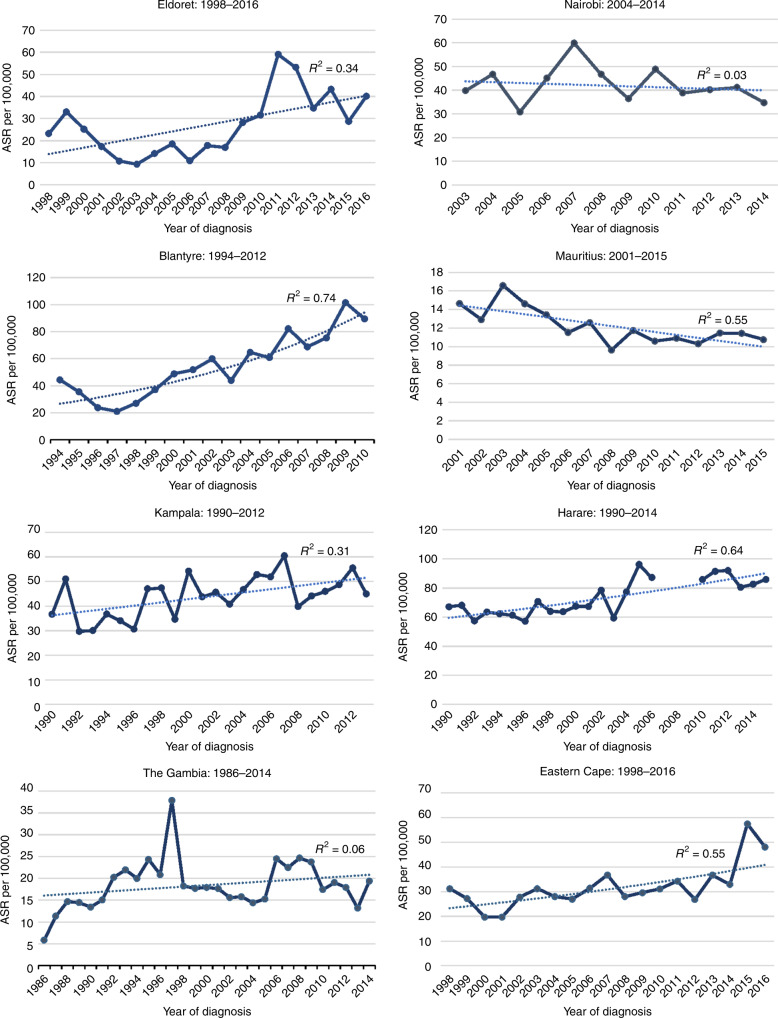
Cervical cancer age standardized incidence rates by year of diagnosis, with best fitting regression line, and corresponding coefficients of determination (R2).

Figure 1 shows the annual ASRs for those registries with data covering time periods of 15 years or more, with the best fitting regression line, and corresponding coefficient of determination (*R*^2^). The values of *R*^2^ ranged from 0.06 in The Gambia to 0.74 in Blantyre. In the graph showing ASRs for Harare, the discontinuation in the line corresponds to the 3-year time period (2007–2009) during which registration was incomplete, and which were therefore excluded from the analyses.

Table 2 shows the results of the Joinpoint analyses for four registries, Nairobi, Kampala, The Gambia and Eldoret, for which the assumption of a single rate of increase was least satisfactory in explaining the time trends over the whole period (*R*^2^ values < 0.5). Joinpoint analyses were used to characterise the trends for these registries. For all four, the data are better explained by two trends. For three registries (Nairobi, Kampala, Gambia), an increase in incidence in the first part of the period (statistically significant in Kampala (AAPC = +2.2%; 95% confidence interval (CI) 0.1; 4.4)) was followed by a (non-significant) decline. For Eldoret, a steep but non-significant decline (until 2002) (−18.3; 95% CI −53.1; 42.4) was followed by a statistically significant increase from 2002 to 2016 (+9.5; 95% CI 3.0; 16.5).

**Table 2:** Joinpoint analyses: time trends in two separate periods.

Registry	Time periods	AAPC^a^ (%)	95% CI of AAPC	*P* value
Eldoret	1998–2002	−18.3	−53.1; 42.4	<0.05
	2002–2016	+9.5	3.0; 16.5	
Nairobi	2003–2007	+7.1	−9.8; 27.1	
	2007–2014	−4.5	−10.0; 1.4	
Kampala	1990–2006	+2.2	0.1; 4.4	<0.05
	2006–2013	−0.7	−5.8; 4.6	
Gambia	1996–1983	+14.6	−1.2; 32.9	
	1983–2014	−1.1	−2.8; 0.5	

^a^Average annual percentage change.

**Fig. 2:**
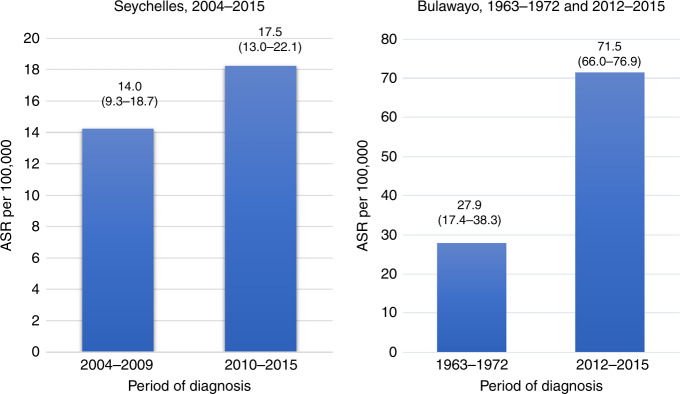
Cervical cancer age standardised incidence rate (ASR) (with 95% confidence intervals) in Seychelles and Bulawayo, by period of diagnosis.

Figure 2 compares ASRs in two time periods in Seychelles (2004–2009 and 2010–2015) and Bulawayo (1963–1972 and 2012–2015). For these two registries, the total number of cases per year were too few to calculate annual rates, and so the results are presented as bar charts by time period. Rates have increased in both, more dramatically in Bulawayo, where they increased more than 2.5-fold in the 50-year period covered.

**Fig. 3:**
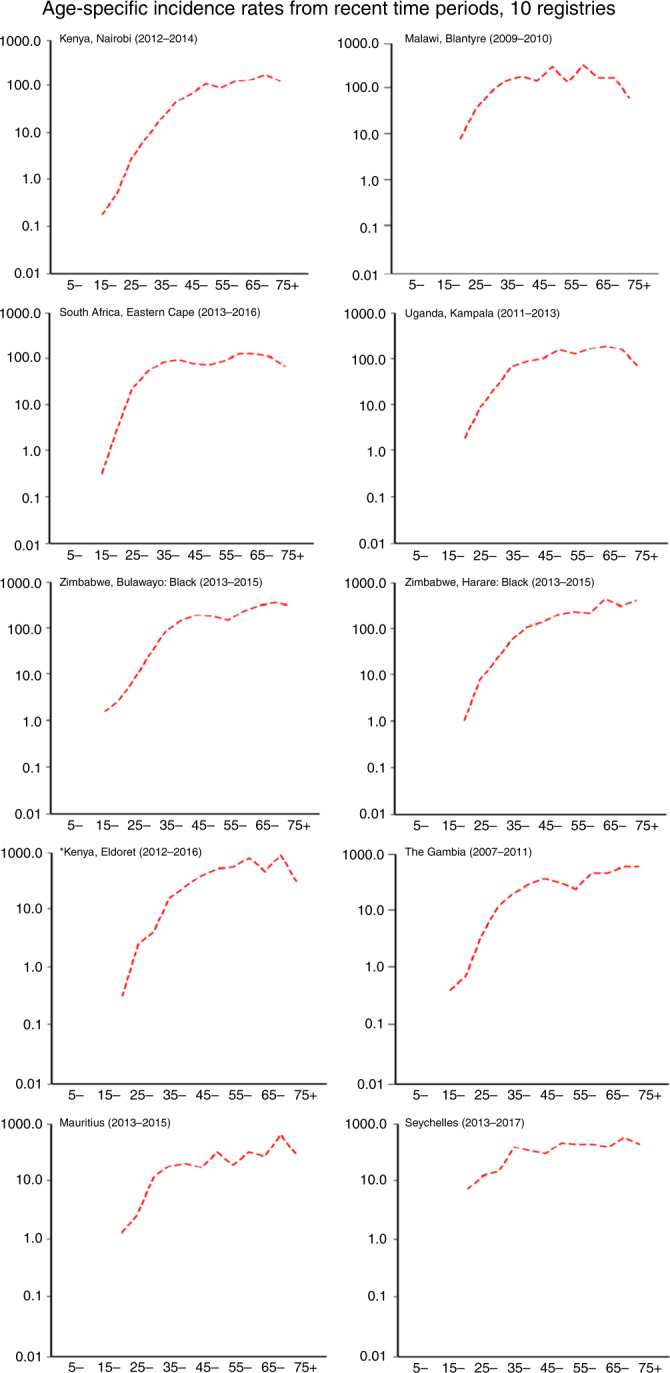
Age-specific incidence rates from recent time periods, 10 registries.

**Fig. 4:**
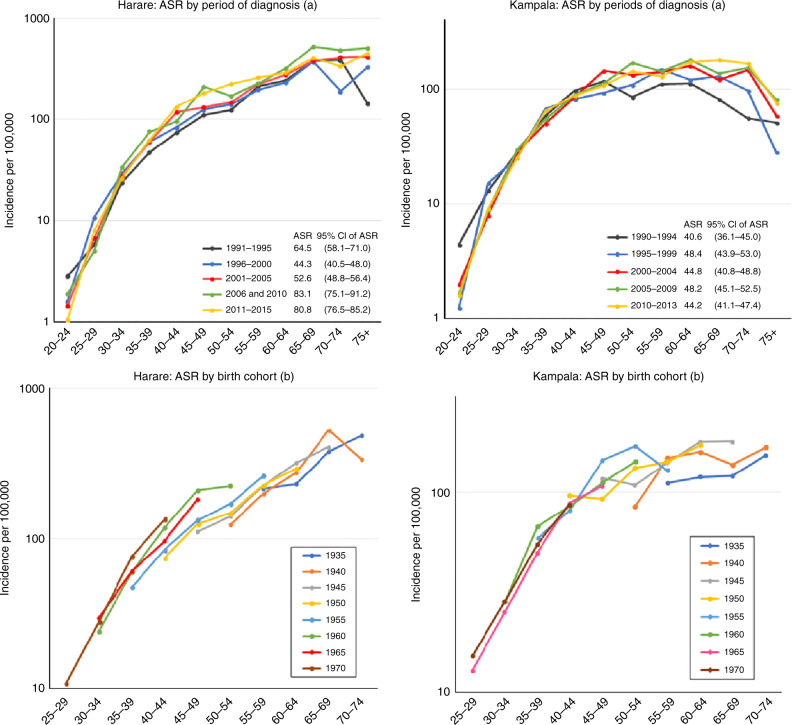
Age-specific incidence rate (ASR) of cervical cancer in Harare and Kampala by period of diagnosis (**a**) and by birth cohort (**b**).

In Fig. 3 we present age-specific rates for the most recent time periods for each of the registries. Across all registries, we observed rapid increases in incidence with advancing age. All the registries reported the highest incidence rates in the 60–64- and 65–69-year age groups, compared with other ages. In some, there is an apparent decline in incidence in the older age groups. This may in part be due to generation-specific increases in the risk of CC, so that rates at a given age are lower in successive birth cohorts. We examined birth cohort-specific trends in Fig. 4, which shows age-specific incidence rates in Kampala (Uganda) and Harare (Zimbabwe) according to both period of diagnosis and birth cohort. The age standardised rates (with 95% CIs) are shown for the 5-year time periods plotted. For Harare, the increase in incidence over time seems to involve all age groups, and time periods (Fig. 4a). When examined by birth cohort (Fig. 4b), there appears to be an increase—at least in the middle-age range—between successive birth cohorts. For Kampala (Fig. 4a) the age-specific trends are less clear, although it does appear that the increases in incidence have involved mainly the older age groups/birth cohorts, with much less change in those born more recently (Fig. 4b). There is no evidence for a decrease in risk of CC with age in either registry when birth cohort-specific rates are examined.

## Discussion

As expected, we found CC incidence rates in eastern Africa to be higher than the rates reported from western and southern Africa, where the breast is the most common site of cancer in women.^21,22^ This variation across these three regions of SSA in part reflects regional differences in the prevalence of chronic HPV infection, the major risk factor for CC,^23^ as well as of HIV, which is known to increase risk in HPV-positive women.^24^ The highest ASRs of CC were reported in Zimbabwe (Harare, and for the most recent time period, Bulawayo), Malawi (Blantyre) and in Uganda (Kampala). The lowest ASRs of CC in our study were reported in Mauritius and Seychelles.

Overall, the findings suggest that CC incidence is on the rise in SSA. While this is clear for Blantyre, Eastern Cape and, in more recent years, the Eldoret cancer registry, the increases elsewhere are smaller and generally not statistically significant. Mauritius appears to be a clear exception, with a persistent and statistically significant declining trend over the years. The Joinpoint analysis suggests a declining incidence in Nairobi and Kampala in more recent years, although the trends are not statistically significant, and for Kampala are compatible with modest increase in incidence (1.3%) over the entire period of observation. It is possible that some of the trends observed represent variation in the quality (completeness) of registration of cancers of the cervix over time. Poor financial support for the activities of most cancer registries in SSA may affect registry operations and lead to incomplete data or reporting delays. However, seven of the registries selected for this study are in the two highest quality categories in IARC’s Globocan estimates programme,^25^ having been published in at least one of the last three volumes of the ‘Cancer Incidence in Five Continents’ series. The data selected for analysis were carefully examined to detect changes in any of the indicators of data quality^19^ over time.

These findings of a high and increasing burden of CC in Eastern Africa are similar to previously published reports.^10,11^ The rising incidence of CC in some countries in the SSA region is in stark contrast to high- and middle-income countries where CC incidence has been on the decline in recent decades.^26^ In these high-income countries, decreases in incidence have largely paralleled the introduction of effective screening programme based on cytology, and have occurred despite high prevalence of persistent HPV infection.^26^ In addition to a rising incidence, previous studies have shown that the majority of cases are diagnosed at late stages^27,28^ (FIGO (International Federation of Gynecology and Obstetrics) III and IV) and in women aged ≥60 years who often have other existing comorbidities, which potentially puts them more at risk of dying from the disease.^29,30^

In SSA where ~90% of all CC cases occur, a high prevalence of HPV,^31^ and human immunodeficiency virus (HIV),^32^ lack of population-wide CC screening programme (and poor uptake where they do exist)^28–30^ and HPV vaccination programmes have contributed to the rising incidence.^14^ It is noteworthy that before the introduction and wide dissemination of Pap testing in the 1960s in the US, the incidence of CC (cumulative risk, 0–74) in ten selected metropolitan areas in 1947–1948 (3.1% in whites and 6.7% in non-whites)^33^ was of the same order of magnitude as the highest rates found in Eastern Africa today. Screening programmes in Africa are generally opportunistic, with low population coverage, or based on visual inspection methods (which have never been demonstrated to lower CC incidence at the population level), or all three.^23,34^

In Mauritius, as part of the country’s national cancer control action plan 2010–2014, a population-wide CC screening program was set up at the Victoria hospital, which provides services to ~30% of the island’s inhabitants. It is possible that this screening program and the country’s transition from a low income to an upper middle-income diversified economy could have contributed to the decline in incidence noted in recent years. The incidence of CC reported in Mauritius is similar to that in other high- and middle-income countries.^21^

Notwithstanding the high morbidity and mortality, CC is a potentially preventable disease with significant implications for public health in SSA, where it accounts for one-quarter of cancer cases and deaths in women.^2^ In 2018, a call to action for coordinated action globally to eliminate CC was made by the Director General of the World Health Organisation (WHO), which has resulted in a new UN Global strategy towards the elimination of CC as a public health problem.^35^ This sets a target of an ASR of 4 per 10^5^ for all countries to achieve within the twenty-first century. Although vaccination against HPV is recognised to be the most effective means of preventing CC, it can have little effect on population-level incidence until the generations of girls vaccinated reach the ages of maximum risk. Therefore, WHO enjoins a comprehensive approach to CC prevention and control, which consists not only of introduction and scaling up of HPV vaccination but also introduction and expanding coverage of screening and treatment of precancerous lesions and prompt management of invasive cancers.

Modelling studies suggest that elimination of cervical cancer is possible in most countries, provided high-coverage screening and vaccination can be achieved, although the likely impact of these interventions indicates that progress will be slowest in low-income countries, as exemplified by almost all of those in SSA.^36^ These projections, as far as Africa is concerned, are based on extremely sparse data on existing trends, and the WHO strategy includes provision for monitoring and surveillance to allow the world to track and improve processes. The draft of the strategy acknowledges^35^ that ‘A fundamental gap among these monitoring and surveillance activities is the lack of population-based cancer registries, which are required to track incidence data… . Together with information on risk factors for non-communicable diseases (provided by population surveys) and mortality (by vital statistics), cancer incidence and survival complete the necessary elements to plan and evaluate the cancer control measures’. It remains to be seen whether this will translate into actual support for the development and maintenance of registries in SSA, the region most hard hit by this disease. We found that only 10 cancer registries in the whole of SSA are available to provide information on the trends of CC in recent years. This is a consequence of inadequate financial support for cancer surveillance. There is therefore an urgent need for government ownership and support for cancer registration in the region, and for international donors to recognise that adequate methods to evaluate the current situation and monitor future trends is an essential component of all cancer control programmes.^37^

## Data Availability

The data that support the findings of this study are available on request. All data requests will be evaluated by the AFCRN research committee. Details of the data application process are outlined on the AFCRN website (http://afcrn.org/index.php/research/how-to-apply/76-research-collaborations).
